# Applying National Estimates of Adults With Indications for Pre-Exposure Prophylaxis to Populations of Men Who Have Sex With Men and People Who Inject Drugs in Colorado: Modeling Study

**DOI:** 10.2196/11113

**Published:** 2019-01-16

**Authors:** Jennifer A Donnelly, Thomas T Deem, Megan A Duffy, Anita K Watkins, Alia A Al-Tayyib, Daniel J Shodell, Mark Thrun, Sarah E Rowan

**Affiliations:** 1 Colorado Department of Public Health and Environment Denver, CO United States; 2 Metro Community Provider Network Denver, CO United States; 3 Denver Public Health Denver Health Medical Center Denver, CO United States; 4 Gilead Sciences Foster City, CA United States

**Keywords:** HIV prevention, key population estimates, men who have sex with men, pre-exposure prophylaxis, people who inject drugs

## Abstract

**Background:**

Oral pre-exposure prophylaxis (PrEP) is a highly effective option for HIV prevention. To realize the full benefit of PrEP at the population level, uptake must reach those at the greatest risk of HIV acquisition. Guidance published by Centers for Disease Control and Prevention (CDC) suggests that the number of individuals with indications for PrEP is 1.1-1.2 million nationally based on survey data of key populations and local transmission patterns. We applied these estimates at state and county levels to determine the number of individuals who might benefit from PrEP locally and compared our estimates to CDC-published estimates for Colorado.

**Objective:**

This analysis aimed to produce estimates of key populations with indications for PrEP in Colorado as a whole and by county type. These estimates will be used for public health strategic planning for HIV prevention goals at the state and county jurisdictional levels.

**Methods:**

Colorado population estimates were obtained from the state demography office, which utilizes US decennial census data and input from county and local agencies to forecast the population. We limited our analysis to adults aged 18-59 years to be consistent with CDC methodology for PrEP estimates. We performed a literature review to define the best population-level percentages to determine numbers of HIV-negative men who have sex with men (MSM) and people who inject drugs (PWID) in Colorado. These percentages were applied to the state and to each county by its rural-urban designation. Finally, CDC-derived percentages of MSM and PWID with indications for PrEP were applied to these estimates to determine numbers of MSM and PWID who may benefit from PrEP use.

**Results:**

In 2017, 3,252,648 adults aged 18-59 years were living in Colorado. By applying published estimates of percentages of men who had sex with other men in the past 12 months, we determined that 41,353-49,624 adult males could be considered sexually active MSM. We estimated that 9758-13,011 adults aged 18-59 years were likely to have injected drugs in the past 12 months. By accounting for numbers of people living with HIV in those categories and applying the CDC PrEP percentages of MSM and PWID with indications for PrEP nationally, we estimated that 8792-12,528 MSM and PWID in Colorado had indications for PrEP; this number is smaller than that estimated by CDC, although within the lower CI limit.

**Conclusions:**

By employing a simple framework consisting of census data, literature review, population estimates, and national estimates for PrEP indicators, we derived estimates for potential PrEP use in our state. Statewide estimates of key populations by state and county type will enable health officials to set informed goals and track progress toward optimizing PrEP uptake. This formula may be applicable to other states with similar epidemics and resources.

## Introduction

Like many states, Colorado observed a decline in HIV diagnosis rates between 2005 and 2015, leading regional stakeholders to consider the possibility of ending the HIV epidemic in the state [1]. The Joint United Nations Programme on HIV/AIDS 90-90-90 movement further propelled that work by introducing international population-level goals for the diagnosis and care of people living with HIV [2]. Colorado is close to reaching the goals of 90-90-90, yet the number of individuals who are newly diagnosed with HIV annually has stabilized and in some regions increased in the past few years, marking a change from the prior decade of declining HIV rates [3]. It is clear that to complement the prevention benefits of optimal treatment for people living with HIV, pre-exposure prophylaxis (PrEP) for people at risk for acquiring HIV is also a necessary tool to end new transmissions and propel efforts to end the epidemic [4].

Unlike the parameters of the 90-90-90 initiative, targets for PrEP use have not been well established at the local level. The National HIV/AIDS Strategy recommends a 500% increase in PrEP prescriptions by 2020, though this is currently considered a developmental indicator, expected to be modified as additional information becomes available [5]. In their paper in the Morbidity and Mortality Weekly Report, Smith et al analyzed survey data from the National Health and Nutrition Examination Survey, National Survey on Drug Use and Health, and National HIV Behavioral Surveillance System and concluded that nationally, approximately 1.2 million individuals in the United States had the Centers for Disease Control and Prevention (CDC)-recommended indications for PrEP [6]. They determined that 24.7% of men who had sex with men (MSM) in the past 12 months, 18.5% of people who injected drugs (PWID) in the past 12 months, and 0.4% of sexually active heterosexual adults had indications for PrEP. A subsequent estimate published in 2018 refined the national estimate to reflect regional differences in HIV transmission risk groups and to identify the number of black, Hispanic or Latino, and white individuals in each transmission group with indications for PrEP in each state. Using this method, CDC reported that 24,310 (95%CI 13,480-44,430) individuals in Colorado have indications for PrEP [7]. To test this estimate and to better understand the need for PrEP in Colorado as a whole and in each county, we utilized a variety of population-level data sources to determine numbers of HIV-negative MSM and PWID in the state who are likely to have indications for PrEP. These estimates were obtained by applying population-level percentages of MSM and PWID to the state as a whole and by calculating county-level estimates based on each county’s rural-urban designation and the percentage of estimated MSM and PWID in each county type. We then applied national CDC-derived percentages of MSM and PWID with PrEP indications to our MSM and PWID estimates to determine the number of individuals with indications for PrEP in Colorado. Our goal was to develop a formula to derive estimates of PrEP indications that could lead to timely, precise, and actionable goals for PrEP uptake at state and local levels.

## Methods

### Colorado Population Estimates

**Figure 1:**
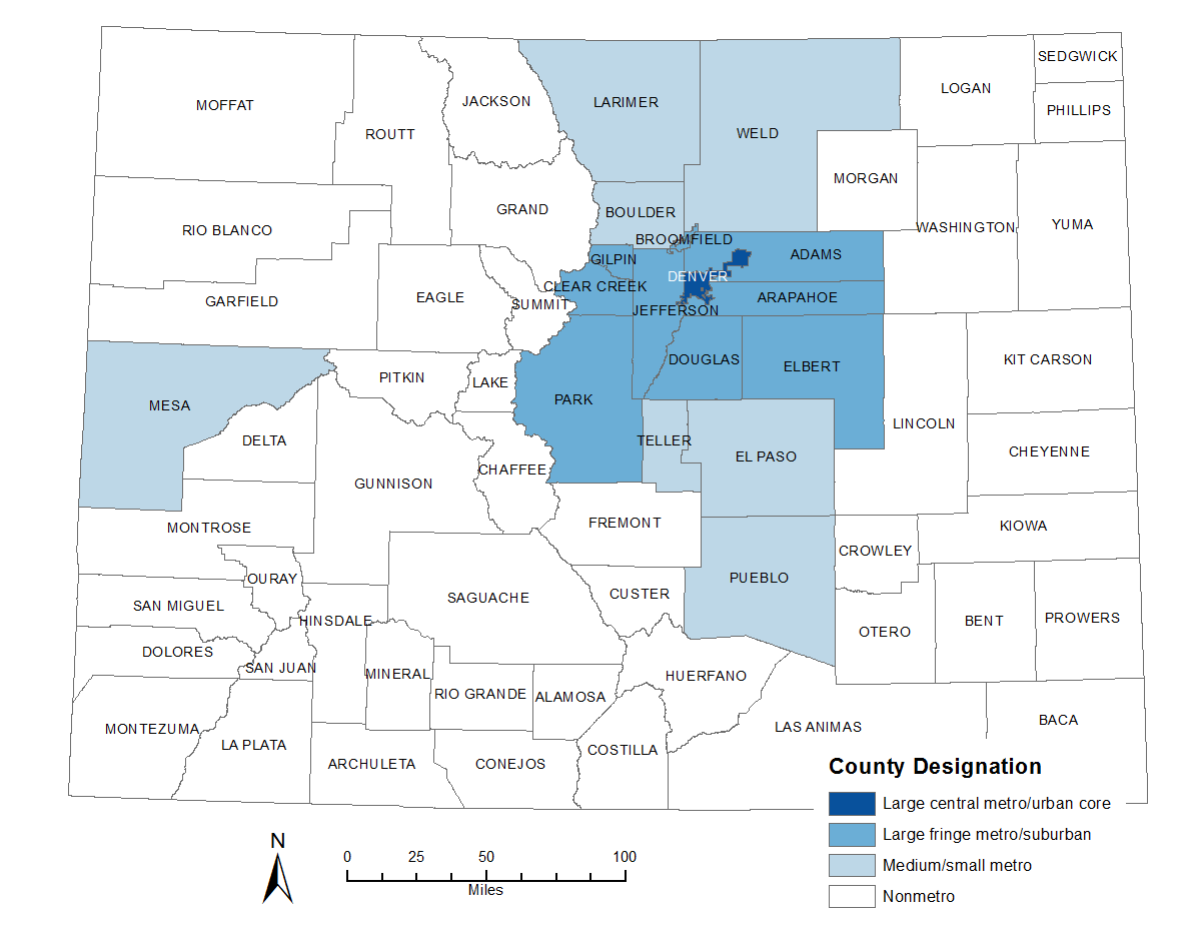
Map of Colorado counties by urbanicity designation.

Census data for the total population of Colorado was obtained from the Colorado State Demography website, which uses the most recent decennial US Census Bureau data and input from county and local agencies to estimate and forecast the population for intercensal years to forecast population at the state and county levels [8]. Numbers of adults aged 18-59 years were extracted from the population totals as was the distribution by sex and the geographic distribution of adults by county. We aggregated Colorado counties using the 2013 National Center for Health Statistics Urban-Rural Classification Scheme for Counties in the following groupings: large central metro/urban core, large fringe metro/suburban, medium/small metro, and nonmetro [9] (see Figure 1). Forecasted 2017 county population data were used to determine total county population, sex, and age stratifications [8].

### Estimates for Men Who Have Sex With Men and People Who Inject Drugs Not Known to Be Living With HIV

We conducted a literature review to determine the most relevant and accurate percentages of adult populations that were likely to fall into the MSM and PWID categories. To reflect populations at increased risk of HIV and to remain consistent with the selection criteria used by Smith and colleagues in their first national PrEP estimates, for our final calculations, we selected references that included estimates of proportions of men who had had sex with other men in the past 12 months and proportions of adults who had injected drugs in the past 12 months [6]. We then applied the national and regional estimates from the literature to the Colorado adult male and overall adult populations. Once the estimated numbers of MSM and PWID in Colorado were calculated, we utilized state HIV surveillance data to subtract the number of individuals known to be living with HIV from each group to determine the potential number of individuals at increased risk of HIV acquisition [3].

**Formulas to determine the number of individuals with indications for pre-exposure prophylaxis (PrEP) by the men who have sex with men (MSM) and people who inject drugs (PWID) subgroups.**

- Estimated number of MSM with PrEP indications=[(Adult male population×Estimated percentage MSM in the past 12 months)−HIV-positive MSM]×Estimated percentage of MSM with PrEP indications (24.7%)
- Estimated Number of PWID with PrEP indications=[(Adult population×Estimated percentage of PWID in the past 12 months)−HIV-positive PWID]×Estimated percentage of PWID with PrEP indications (18.5%)

Individuals with a history of both male-male sex and injection drug use were subtracted from the MSM estimates. The exercise was repeated at the county level by applying varying percentages of individuals estimated to have had male-male sex or injected drugs in the past 12 months by county type (urban-rural designation) and then subtracting the number of MSM or PWID living with HIV in each county type from the total.

### Estimates for Men Who Have Sex With Men and People Who Inject Drugs With Indications for Pre-Exposure Prophylaxis

Using CDC-derived percentages of individuals in the MSM and PWID categories with indications for PrEP, we calculated the number of individuals in these categories by applying percentages to Colorado as a whole and by summing estimates for each county type [6] (see Textbox 1 for the complete formula).

## Results

### Colorado Population Estimates

The number of adults aged 18-59 years living in Colorado in 2017 was 3,252,648. Of those, 50.58% (1,654,138/3,252,648) were men. Overall, 52.66% (1,713,125/3,252,648) of the population lived in counties classified as large central metro/urban core or large fringe metro/suburban[8] (see Table 1 for the distribution of adults by county urbanicity type).

**Table 1:** Distribution of Colorado population in 2017 by urbanicity.

Urbanicity type		Counties	Male population	Female population	State population
State, n (%)		64 (100.00)	1,654,138 (100.00)	1,598,510 (100.00)	3,252,648 (100.00)
**Urbanicity^a^, n (%)**					
	Large central metro/urban core	1 (1.56)	228,148 (13.79)	220,601 (13.80)	448,749 (13.79)
	Large fringe metro/suburban	9 (14.06)	633,801 (38.32)	630,575 (39.45)	1,264,376 (38.87)
	Medium/small metro	7 (10.93)	585,451 (35.39)	568,440 (35.56)	1,153,892 (35.47)
	Nonmetro	47 (73.43)	206,737 (12.50)	178,894 (11.19)	385,631 (11.85)

^a^Counties assigned to urbanicity in accordance with the 2013 National Center for Health Statistics urban-rural classification scheme.

### Estimates for Men Who Have Sex With Men and People Who Inject Drugs Not Known to Be Living With HIV

Our literature review yielded 4 publications that characterized the percentage of the given populations of males that could be considered MSM, 3 of which included estimates for male-male sexual activity in the past 12 months [10-13]. The review yielded 2 publications that described population proportions of PWID, 1 of which specifically characterized the percentage of populations with a history of injection drug use in the past 12 months [11,14] (see Table 2 for details of the reviewed publications). The estimates most applicable to our analysis were described by Oster et al [11], who suggested that 2.5% of the male population nationally and 3% of the males in the Western United States had a history of sex with men in the past 12 months. At the county level, estimates for recent male-male sexual activity ranged from 1.1% of adult males in nonmetro counties to 4.4% of adult males in large central metro counties [11]. We compared these findings with estimates produced by Grey et al [10], who suggested that 2.4% of the adult male population nationally had a history of sex with men in the past 12 months and that 3.8% of men in Colorado had had sex with a man in the past 5 years. Male-male sexual activity in Colorado in the past 12 months was not described in the study by Grey et al. County-level estimates ranged from 1% to 6.8% in nonmetro and large central metro counties, respectively [11].

Oster et al [11] also estimated that 0.3% of the adult population nationally and 0.4% of the adult population in the Western United States had a history of injection drug use in the past 12 months. Estimates of recent injection drug use at the county urbanicity levels ranged from 0.3% in the large central metro counties to 0.5% in the nonmetro counties [11].

By applying the national and regional MSM and PWID percentages by Oster et al [11] to the Colorado population as a whole, regardless of county type, we determined that statewide, 41,353-49,624 males aged 18-59 years were likely to be MSM in the past 12 months, depending on the whether we applied the national MSM estimates (lower estimate) or Western US MSM estimates (higher estimate). Using the national and Western US estimates for PWID, we determined that 9758-13,011 individuals (males and females aged 18-59 years) were likely to have injected drugs in the past 12 months, also with the higher estimate derived from estimates for the Western United States rather than nationally. After accounting for individuals living with HIV in those 2 categories, we determined that 33,199-41,470 MSM and 9098-12,351 PWID were eligible for the PrEP indications analysis.

When we applied estimates of percentages of individuals with MSM and PWID behavior in the past 12 months by county urbanicity type to the number of adults ages 18-59 years living in each county type in Colorado, we determined that 36,354 males were estimated to have had male-male sex in the past 12 months and 10,143 individuals were likely to have injected drugs in the past 12 months. After accounting for MSM and PWID living with HIV in Colorado, we estimated that 28,200 MSM and 9483 PWID were eligible for the PrEP indications analysis.

### Estimates for Men Who Have Sex With Men and People Who Inject Drugs With Indications for Pre-Exposure Prophylaxis

**Figure 2:**
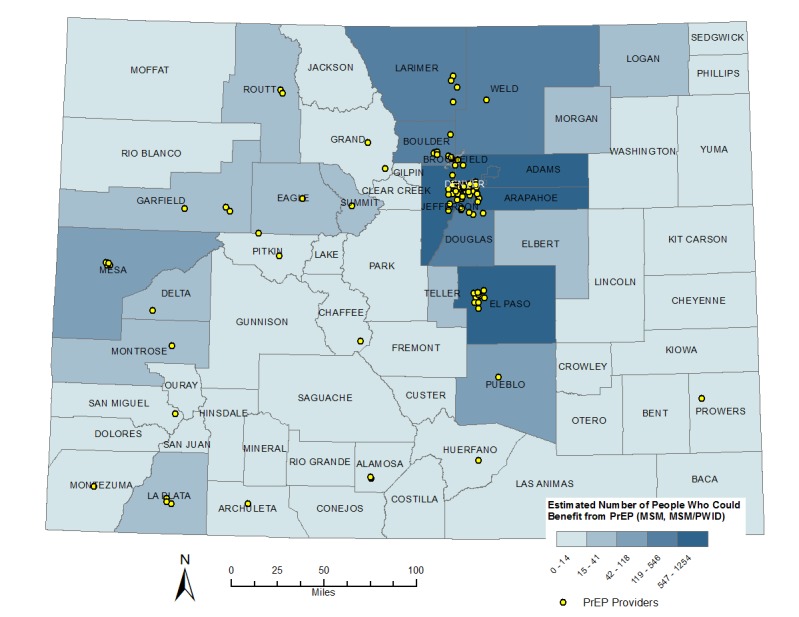
Map of Colorado counties with numbers of men who have sex with men (MSM), including MSM that also inject drugs (MSM/PWID) with indications for pre-exposure prophylaxis (PrEP).

**Figure 3:**
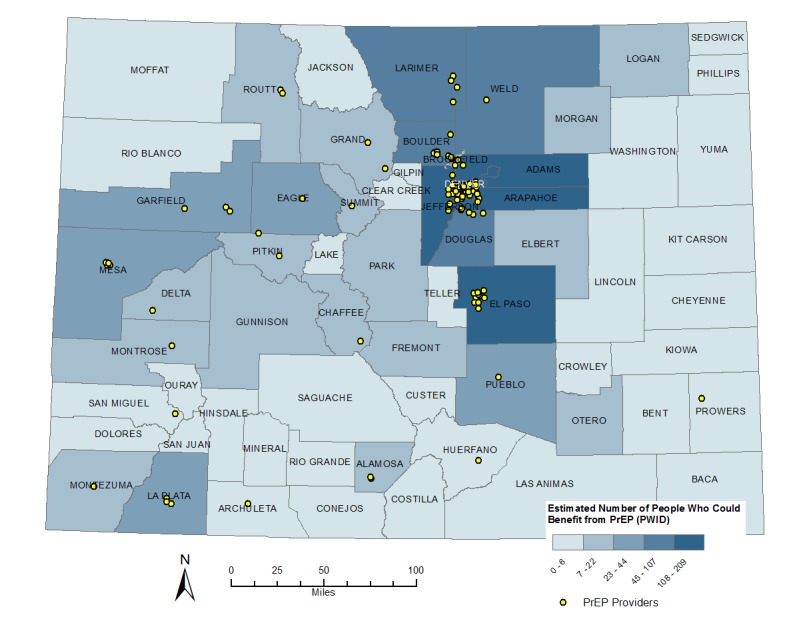
Map of Colorado counties with numbers of people who inject drugs (PWID) with indications for pre-exposure prophylaxis (PrEP).

Applying published estimates for the proportions of MSM and PWID with CDC-recommended indications for PrEP, we determined that the MSM population with indications for PrEP ranged from 8200 to 10,243 males statewide. We estimated that 1683-2285 PWID had indications for PrEP statewide. By applying the formula for PrEP indications using the county-level MSM and PWID estimates, we determined that 6965 MSM in Colorado were likely to have indications for PrEP and 1827 PWID were likely to have indications for PrEP (see Table 3 for the estimated numbers of MSM and PWID with indications for PrEP statewide and by county type). Majority of MSM with PrEP indications were located in a large central metro county (Denver) or in large fringe metro counties, while the number of PWID with PrEP indications was more evenly distributed throughout the state (see Figures 2 and 3).

**Table 2:** Literature reviewed for population estimates [10-14].

Study and behavioral characteristic		Time frame	US population percentage^a^, n (%)	Colorado or Western US population percentage, n (%)	Population age (years)	Geographic distribution
**Oster et al, 2015**						
	MSM^b^	Lifetime	5,933,000 (4.7)	1,696,000 (5.7)	≥13	National, regional, and county level
	MSM	Past 12 months	3,156,000 (2.5)	893,000 (3.0)	≥13	National, regional, and county level
	PWID^c^	Lifetime	5,949,000 (2.3)	1,980,000 (3.3)	≥13	National, regional, and county level
	PWID	Past 12 months	776,000 (0.3)	240,000 (0.4)	≥13	National, regional, and county level
Grey et al, 2016: MSM		Past 5 years	4,503,080 (3.9)	73,357 (3.8)	≥18	National, state, county, and core-based statistical areas
**Purcell et al, 2012**						
	MSM	Lifetime	8,476,848 (6.9)	N/A^d^	≥13	National
	MSM	Past 5 years	4,791,262 (3.9)	N/A	≥13	National
	MSM	Past 12 months	3,562,733 (2.9)	N/A	≥13	National
Lieb et al, 2011: MSM		Lifetime	12,986 (6.9)	N/A	≥18	State level
Lansky et al, 2014: PWID		Lifetime	6,612,488 (2.6)	N/A	≥13	National level

^a^MSM: percentage of adult males; PWID: percentage of all adults.

^b^MSM: men who have sex with men.

^c^PWID: people who inject drugs.

^d^N/A: not applicable.

**Table 3:** Estimated number of individuals with indications for pre-exposure prophylaxis in Colorado.^a^

Key population	Statewide (United States based)^a^	Statewide (Western United States based)	Statewide (sum of county-level estimates)	Large central metro	Large fringe metro	Medium/ small metro	Nonmetro
Men who have sex with men, n	8200	10,243	6965	1536	3311	1676	442
People who inject drugs, n	1683	2285	1827	207	669	610	341
Total, n	9683	12,528	8792	1743	3980	2286	783

^a^This table presents only “n” values. All estimates were derived by applying the CDC percentages for PrEP in MSM (24.7%) and PWID (18.5%) across all estimates.

## Discussion

### Principal Findings

Of the 3,252,648 individuals aged 18-59 years residing in Colorado in 2017, we determined that 8792-12,528 MSM and PWID were likely to have indications for PrEP as described in the 2015 CDC guidelines for PrEP use. Of those, approximately 81%-82% were MSM, and 65% lived in large central or large fringe metro areas. By target population, 70% of MSM lived in large central or large fringe metro areas and 50% of PWID lived in large central or large fringe metro areas, reflecting the more rural distribution of injection drug use. The county-level distribution of PrEP for MSM was similar to the distribution of HIV among MSM in Colorado, in which 70%-75% of the MSM living with HIV resided in the 5-county Denver Metro area [3].

As has been observed with national PrEP estimates, the number of individuals estimated to have an indication for PrEP in Colorado was close to the number of individuals living with HIV in the state [6]. Some authors have suggested that an alternate method for estimating PrEP need could employ HIV diagnoses as a reference point [15]. In 2016, approximately 8500 MSM, 1400 MSM-PWID, and 500 PWID were known to be living with HIV in Colorado, which are similar number overall to the estimates for PrEP we present here [3]. However, the number of non-MSM-PWID living with HIV in Colorado is significantly lower than the number of individuals we estimated were PWID with indications for PrEP.

A more nuanced approach to using HIV diagnosis data to estimate numbers of individuals with indications for PrEP at the local level was recently published by the CDC. This approach relies on the ratio of the percentage of PWID diagnosed with HIV to the percentage of MSM diagnosed with HIV in a given area. This ratio is further refined by applying race and ethnicity data [16]. This approach accounts for heterogeneity in transmission risk factors regionally. Interestingly, the published estimates for MSM with PrEP indications in Colorado were significantly higher than those we present here. This is partly due to the weighted proportion of HIV diagnoses in Colorado who are MSM, but more importantly, this more recent publication relied on much larger MSM estimates based on the report of sexual activity with men in the past 5 years as opposed to that in the past 12 months, as is in the PrEP guidelines [7]. This significant methodological difference is the key to understanding the variation in estimates. Given that sexual activity varies for people over time, especially in the light of advances in PrEP and understanding of treatment as prevention, having both estimates gives a broader picture of the potential for PrEP use in the state. This alternative methodological approach is limited in states with lower numbers of HIV diagnoses annually, where the transmission category distribution may vary significantly year to year. The recent CDC publication also does not describe estimates for numbers of individuals with PrEP indications at the county level, which are crucial for local resource allocation and planning, in particular as it relates to support for PrEP clinical services in underserved counties (see Figures 2 and 3). The method we present here enables jurisdictions to generate targeted PrEP estimates based on more timely local data, which can then be compared to nationally generated state-level estimates as they become available.

### Limitations

Our analysis is subject to several limitations. Most notably, we did not include estimates of heterosexual individuals with indications for PrEP. We initiated this process using similar methodology as that employed for MSM and PWID estimates but deemed the estimates likely to be inaccurate as the epidemiology of HIV in Colorado is heavily skewed toward MSM with a much lower percentage of individuals living with HIV in the state being heterosexual than is the case nationally. Similarly, we were not able to estimate the prevalence of transgender people in Colorado or subsequent numbers of transgender individuals with indications for PrEP. The inclusion of these 2 populations would make the analysis richer and more complete and will be the focus of future efforts at the state and local health department levels.

The analysis is also limited by our reliance on national and regional estimates of sexual behavior and injection drug use, which may or may not be accurate for Colorado. In particular, Colorado has been heavily affected by the opioid epidemic and may have a significantly higher number of PWID than presented here [17]. Also, both the behavioral estimates obtained from the papers by Grey et al and Oster et al as well as the PrEP indication estimates by Smith et al rely on data from the National Health and Nutrition Examination Survey, which is limited to individuals who are not institutionalized or homeless [6,10,11]. This exclusion is likely to lead to an underestimation of the prevalence of recent injection drug use, thereby leading to an underestimate of the number of PWID with indications for PrEP. A revised estimate of the prevalence of PWID that accounts for homeless and incarcerated populations would be of great benefit. Finally, as noted by Smith and colleagues, as sample sizes get smaller, estimation is more unstable [6]. Therefore, the estimates we have presented have been used specifically for program planning purposes and are limited in generalizability.

### Implications and Final Summary

Although simple in its methodology, this exercise is a practical means to estimate the need for PrEP at the state and local levels. To our knowledge, this is the first instance of a state- and county-level application of national estimates. Additional methodologies using surveys and more precise population-level statistics have been employed in other jurisdictions as alternate approaches to estimating PrEP demand or PrEP targets [18-20]. However, to obtain preliminary estimates, especially for states with relatively smaller epidemics or for whom resources for population-level HIV prevention analyses are more limited, we offer this approach as a feasible option that provides immediate, actionable estimates that can be quickly revised as new estimates for key populations become available.

As Colorado and its individual metro areas develop strategic plans to end the HIV epidemic, measurable targets for PrEP uptake help direct efforts to the most relevant populations and regions [1]. While the estimates for PrEP indication vary when derived by county type compared to the statewide estimates we produced and compared to the most recent estimates for Colorado from CDC, taken as a whole, these estimates provide a range of numbers that can serve as targets for PrEP use, possibly in a stepwise manner starting with more conservative estimates and increasing our targets as we build demand and gain capacity for PrEP provision. At this time, our state health department is conducting an analysis of insurance claims data to determine the approximate number of PrEP prescriptions filled in 2017. This will serve as a starting point for measuring progress toward optimal PrEP uptake. Ongoing education and financial support for PrEP programs will be crucial to ensuring that this highly effective intervention reaches all individuals for whom it could be beneficial.

## Abbreviations

CDC: Centers for Disease Control and Prevention
MSM: men who have sex with men
PrEP: pre-exposure prophylaxis
PWID: people who inject drugs
